# Prebiotic capacity of novel bioengineered wheat arabinoxylans in a batch culture model of the human gut microbiota

**DOI:** 10.3389/frmbi.2023.1156797

**Published:** 2023-06-14

**Authors:** Emmanuel N. Njoku, Walid Mottawea, Hebatoallah Hassan, Riadh Hammami

**Affiliations:** ^1^ School of Nutrition Sciences, Faculty of Health Sciences, University of Ottawa, Ottawa, ON, Canada; ^2^ Department of Microbiology and Immunology, Faculty of Pharmacy, Mansoura University, Mansoura, Egypt; ^3^ Department of Biochemistry, Microbiology and Immunology, Faculty of Medicine, University of Ottawa, Ottawa, ON, Canada

**Keywords:** gut microbiome, enzymatic bioengineering, wheat arabinoxylan, prebiotics, prebiotic effect, short-chain fatty acids, *in vitro* fermentation

## Abstract

Arabinoxylan (AX) is an essential component of dietary fiber with potential prebiotic properties. However, owing to its complex structure, fermentation of AX by gut microbes is structure dependent. In this study, we evaluated the effect of bioengineered wheat AX on the metabolism and composition of gut microbiota using an *in vitro* fermentation model. We compared the effect of bioengineered AX with that of untreated AX and a control. Structurally modified AX did not significantly alter gut microbiome composition within 48 h of treatment; however, it enhanced the abundance of health-promoting bacterial taxa, such as *Bacteroides*, *Bifidobacterium*, *Anaerofustis*, and *Eubacterium.* Furthermore, the bioengineered AX significantly increased the level of acetate produced over 24 h. The amount of microbiota-generated butyrate was significantly increased 24 h after adding α-L-arabinofuranosidase-treated AX. AX treated with the α-L-arabinofuranosidase B25 enzyme induced higher levels of production of total short-chain fatty acids by the microbiota from four donors. The results of this study provide evidence that enzymatic structural modification of AX has the potential to modulate gut microbiome composition and metabolic activities.

## 1 Introduction

The human gut is home to a highly diverse ensemble of microorganisms, comprising approximately 10^14^ microbial cells from more than 1,000 species, and contains nearly 100 times as many genes as the human genome (Qin et al., 2010; Demuth et al., 2021). It has enormous metabolic capability (Putignani et al., 2014), and, hence, it plays a significant role in an individual’s health and overall well-being. Microbial dysbiosis refers to a change in the composition of the human gut microbiota, from a generally diversified and commensal microbial community to a more maladaptive and pathogenic profile (Patterson et al., 2014; Keightley et al., 2015). For example, using some common antidepressants and having poor dietary feeding patterns can negatively alter the gut microbiome composition and reduce the production of beneficial metabolites by gut microbes (Ait Chait et al., 2020; Beam et al., 2021). Furthermore, Jones and colleagues characterized dysbiosis as an increase in the population of adherent and/or invasive *Escherichia coli*, and a decrease in *Bacteroidetes* and *Firmicutes* phyla, including the clinically meaningful *Faecalibacterium prausnitzii* (Jones et al., 2014).

Dietary fibers are one of the core components of food that strongly influence the composition and activity of the gut microbiome (Romero Marcia et al., 2021). Hence, ingesting dietary fiber is key to a healthy diet and diverse gut microbiome (Demuth et al., 2021). In addition, recent dietary intervention studies have explored diet as an avenue to modulate the composition and function of gut microbes in the gastrointestinal tract (Li et al., 2019; Rinninella et al., 2019; Mottawea et al., 2020). However, it is essential to note that not all dietary fibers are prebiotics. To be considered a prebiotic, dietary fiber must elicit certain health benefits for the host and support the growth of beneficial gut microbes (Gibson et al., 2017). In addition to modulating the gastrointestinal tract and enhancing the availability of beneficial gut microbes, prebiotic dietary fibers are important for improving the metabolic profile of the gut microbiome. Numerous studies have revealed that short-chain fatty acids (SCFAs) are produced by the fermentation of prebiotic dietary fibers by human colonic microbes (Fehlbaum et al., 2018; Marín-Manzano et al., 2020; Demuth et al., 2021; Zhu et al., 2022). These SCFAs regulate metabolic syndrome, serve as important energy sources for colonic epithelial cells, and confer health benefits to the host. Acetate, butyrate, and propionate are the most dominant SCFAs produced by the gut microbiota during the fermentation of prebiotic fibers (Fu et al., 2018; Ding et al., 2019).

Arabinoxylan (AX) is one of the most common dietary fibers available in various cereals, including wheat, corn, and millet sorghum (Hughes et al., 2007). Wheat remains one of the primary sources of daily dietary fiber consumption in Western diets (Stevenson et al., 2012), and is widely accessible at a moderate cost (Deroover et al., 2020). Furthermore, AXs have garnered considerable attention owing to their potential prebiotic properties, which can be affected by their complex structural features (Sun et al., 2019). Rose and colleagues have also identified wheat AX as a candidate prebiotic (Rose et al., 2010). Several studies have revealed that slight structural differences in prebiotic dietary fibers can affect gut microbiota (Deehan et al., 2020; Tuncil et al., 2020), and these structural variations may target certain microbial species (Louis, 2017; Cantu-Jungles and Hamaker, 2020), thus affecting the production of SCFAs. Although AX has some favorable health-promoting properties, it is still uncertain how its enzymatic structural modification influences human gut microbiota and SCFA production.

**Figure 1:**
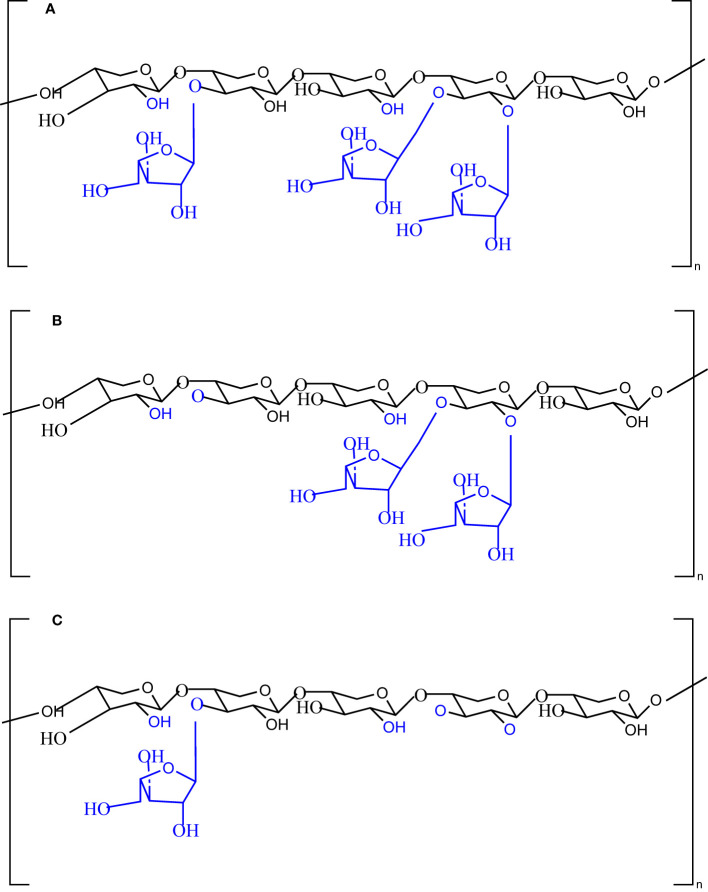
Chemical structures of the native **(A)** and modified **(B, C)** arabinoxylan polymers with the potential catalytic sites of the enzyme α-L-arabinofuranosidase B25 from *Bacteroides ovatus* and α-L-arabinofuranosidase from *Bifidobacterium adolescentis*.

In the present study, we investigated the impact of enzymatic structural modification of wheat AX on the composition and metabolism of the human gut microbiome using batch culture and an *in vitro* fermentation model. Two carbohydrate-active enzymes (arabinofuranosidases) were used for this purpose. First, α-L-arabinofuranosidase B25 from *Bacteroides ovatus* and α-L-arabinofuranosidase from *Bifidobacterium adolescentis* were used for the structural modification of the AX fiber. As a result of the enzymes targeting specific catalytic sites on AX, distinct polymers of AX are produced, as shown in **Figure 1**. Individual treatment of AX with α-L-arabinofuranosidase B25 produces only L-arabinofuranosyl units on double-substituted β-D-xylopyranosyl units (**Figure 1B**), whereas treatment with α-L-arabinofuranosidase produces only L-arabinofuranosyl units on single-substituted β-D-xylopyranosyl units (**Figure 1C**). Both untreated and treated AX were subjected to *in vitro* fecal fermentation for 48 h, and the microbiota composition was assessed using 16S ribosomal RNA (rRNA) sequencing, whereas metabolic activity was evaluated by quantifying SCFAs produced using gas chromatography.

## 2 Materials and methods

### 2.1 Arabinoxylan fibers, enzymes, and standards

Wheat AX with a high (cat no. P-WAXYH, viscosity 56 cSt; hereafter termed HAX), medium (cat no P-WAXYM; viscosity 31 cSt; hereafter termed MAX), and low (cat no P-WAXYL; viscosity: 13 cSt; hereafter termed LAX) molecular weights, along with α-L-arabinofuranosidase B25 enzyme (product code E-ABFBO25) from *Bacteroides ovatus*, with an optimum temperature of 40°C, and α-L-arabinofuranosidase enzyme (product code: E-AFAM2) from *Bifidobacterium adolescentis*, with an optimum temperature of 40–50°C, were purchased from Megazyme International (Ireland). The internal standard 2-ethylbutyric acid and the external standard volatile-free acid mix used for SCFA analysis were purchased from MilliporeSigma (Oakville, ON, Canada).

### 2.2 Arabinoxylan fiber dissolution and enzyme digestion

A total of 0.3 g of wheat AX fiber (LAX, MAX, or HAX) was weighed and added to a sterilized conical flask. To ensure complete fiber dissolution, 2.4 mL of 95% ethanol was added, followed by 27 mL of sterilized water, and the fiber was stirred using a magnetic stirrer at 100°C until complete dissolution. The solution was allowed to cool, and the volume was adjusted to 30 mL. Subsequently, 200 μL of α-L-arabinofuranosidase B25 was added to obtain fibers with double-substituted xylose units, and 200 μL of α-L-arabinofuranosidase was added to obtain fibers with single-substituted xylose units. The samples were incubated at 40°C for 24 h, followed by termination of the enzymatic reaction by heating the samples at 95°C for 5 min and then centrifugation at 7,400 rpm for 10 min. The resulting supernatants were transferred to test tubes and stored for *in vitro* fecal fermentation experiments.

### 2.3 Fecal sample collection and cell immobilization in gel beads

Fecal samples were obtained from four healthy adult donors (two males and two females) who did not have any known medical conditions, and had not been exposed to antibiotic treatment, antidepressant treatment, or probiotic or prebiotic supplements for at least 3 months prior to sample collection. The collection of fecal samples was approved by the University of Ottawa Research Ethics Board and Integrity (ethics file number: H-02–18–347; approval date 5 March 2018). The feces were processed into slurries by dilution in reduced peptone water [20% weight per volume (w/v; weight to volume)], homogenized, and further immobilized in 1- to 2-mm gel beads consisting of gellan gum (2.5% w/v), xanthan (0.25% w/v), and sodium citrate (0.2% w/v) under anaerobic conditions, as described previously (Mottawea et al., 2020). The immobilized microbial population from each donor was used to inoculate one bioreactor that was run in continuous fermentation mode for 12 days to develop a stable and highly diverse microbiome community.

### 2.4 Experimental setup and fermentation procedure

#### 2.4.1 Culture medium

MacFarlane broth was used as the nutrient medium in this experiment. MacFarlane broth is a complex nutritional medium that closely replicates the nutrients found in the large intestine of healthy adults (Macfarlane et al., 1998).

#### 2.4.2 *Ex vivo* development of the fecal microbiome community

Continuous fermentation was carried out for 12 days using an *ex vivo* model mimicking the human proximal colon (N*u*GUT Research Platform, University of Ottawa), as previously described (Mottawea et al., 2020; Mousavi et al., 2022). The experimental setup consisted of four bioreactors. Each reactor was set up to mimic the microbiological and physiological conditions of the adult proximal colon (pH 5.7, with stirring at 120 rpm, at 37°C, and a mean retention time of 8 h). Anaerobiosis was ensured through continuous headspace flushing with dinitrogen (N_2_) and carbon dioxide (CO_2_) at a ratio of 0.9 : 0.1, while the addition of sodium hydroxide (NaOH) (2.5 M) maintained a constant pH of 5.7. The fermentation process was initiated by inoculating 60 mL of immobilized gel beads into each of the four bioreactors containing 140 mL of newly prepared sterile MacFarlane culture medium, as previously described (Mousavi et al., 2022). The colonic model was run in batch culture fermentation mode for 48 h to enhance bead colonization. After 48 h, the colonic model was switched to continuous mode for the remaining 12 days for microbiota stabilization. Subsequently, the developed microbial community was employed for *in vitro* testing of the impact of the treated and untreated AX fibers.

#### 2.4.3 *In vitro* fecal fermentation of treated and untreated arabinoxylan

**Figure 2:**
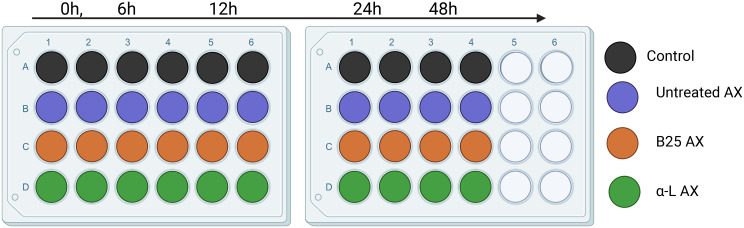
Experimental setup of *in vitro* fecal fermentation for 48h.

The microbiota was derived from four independent bioreactors containing microbiota from four different individuals. *In vitro* fecal fermentation was performed using MacFarlane medium (Macfarlane et al., 1998). MacFarlane medium without fibers was prepared as described by Demuth et al. (2021), with slight modifications. The tested AX concentration (5 mg/mL) was calculated based on the usual Western human adult fiber intake (i.e., 15 g/day). The mixture of AX and the medium solution was added to 24 well plates, and inoculated with the cultivated microbiota at an inoculation rate of 1% (v/v; volume to volume) and a final fermentation volume of 2 mL. The plates were incubated anaerobically at 37°C for 48 h. The samples (2 mL) were collected at 0, 6, 12, 24, and 48 h. The collected samples were separated by centrifugation at 14,000 g for 5 min at 4°C. The pellet was used for metagenomic DNA extraction, and the supernatant was used to determine the presence of SCFAs. The fermentation experiment was conducted in duplicate for each treatment per donor and per time point (**Figure 2**).

### 2.5 Microbial community analyses

#### 2.5.1 Genomic DNA extraction

Following the manufacturer’s instructions, genomic DNA was extracted from the pellet of fecal slurry and fermentation samples using the Fast DNA Spin Kit (MP Biomedicals, Solon, OH, USA), and mechanical lysis was performed using a Bead Mill-24 homogenizer (Fisher Scientific, Ottawa, ON, Canada), as previously described (Mottawea et al., 2020). The quantity of extracted DNA was measured using a Qubit™ fluorometer (Invitrogen, Carlsbad, CA, USA) and stored at −20°C until further analysis.

#### 2.5.2 16S metagenomic sequencing

Microbial community composition and diversity of the fecal slurry and fermentation samples were evaluated using 16S rRNA gene-based MiSeq sequencing (Illumina, San Diego, CA, USA). The V3–V4 regions of the 16S rRNA gene were amplified using dual-barcoded primers, and an amplicon library for sequencing was constructed using the Illumina standard protocol. The amplicon libraries were pooled in equimolar amounts and paired-end sequenced using the Illumina MiSeq platform (N*u*GUT Research Platform, University of Ottawa) and the MiSeq Reagent Kit v3 (600-cycle) (Illumina) in accordance with the standard protocol.

#### 2.5.3 Metagenomic sequencing data analysis

The generated sequences were processed using the QIIME 2.2020.8 pipeline (Caporaso et al., 2010). Initially, sequences were quality filtered and denoised using the DADA2 pipeline (Callahan et al., 2016), and clustered into observed features based on 97% similarity using the Greengenes database (v13.8). The observed features were rarefied into an equal number of 20,000 reads per sample using QIIME. Shannon entropy was calculated to compare alpha diversity. Beta diversity among samples was determined using the Bray–Curtis distance and visualized using principal coordinate analysis (PCoA). Permutational multivariate analysis of variance and 999 permutations (Bolyen et al., 2019) were employed to assess the contribution of different factors to gut microbiota diversity. To identify significantly different taxa, the relative abundances of each taxon were summed, normalized, and autoscaled. A linear mixed model was then used to identify taxa with differential abundance by incorporating donor and time as confounding covariates. The Kruskal–Wallis test followed by the two-stage Benjamini, Krieger, and Yekutieli false discovery rate procedure was used for statistical analysis when needed.

### 2.6 Quantification of short-chain fatty acids

The concentrations of SCFAs, including butyric, acetic, and isovaleric acids, were assessed by gas chromatography with a flame ionization detector (Mottawea et al., 2020). All samples were analyzed twice (two technical measures). External standards (MilliporeSigma) were used for peak identification and quantification, and the data were expressed as the concentration of SCFAs in mM.

### 2.7 Statistical analysis

Data from gas chromatography analyses were analyzed using GraphPad Prism v8.3. (GraphPad Software, San Diego, CA, USA) to assess the significance of the results among treatments. Statistical comparisons were conducted among different treatments simultaneously using repeated measures of two-way analysis of variance, followed by Tukey’s multiple comparisons test (*p* < 0.05). Statistical comparisons of metagenomic sequencing data are presented in Section 2.5.3.

## 3 Results

### 3.1 Gut microbiota diversity

**Figure 3:**
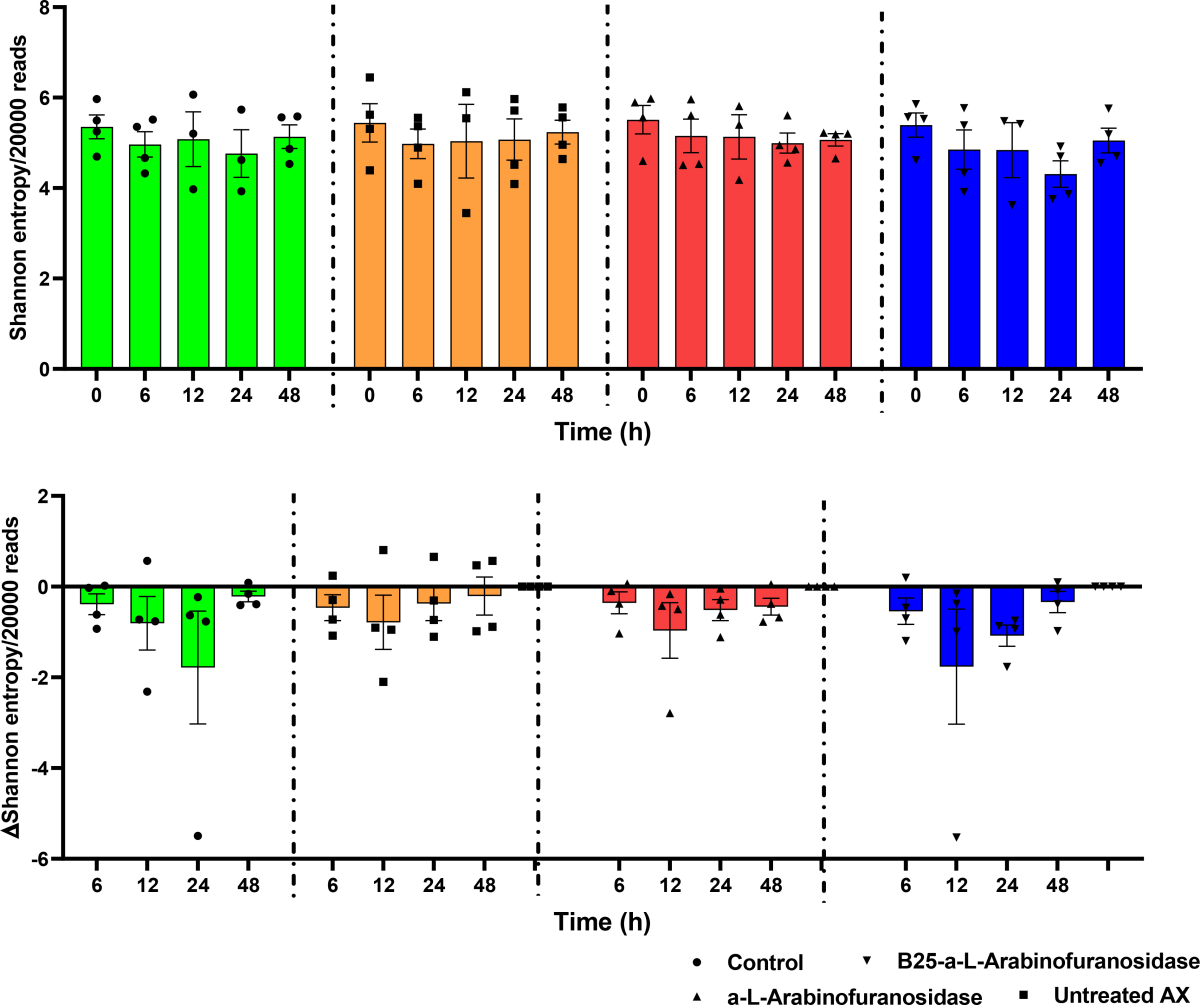
Shannon entropy of the identified microbiota from microplates with no-treatment control, α-L-arabinofuranosidase B25 enzyme-treated AX, α-L-arabinofuranosidase enzyme-treated AX, and untreated AX at different time intervals. The resulting data were analyzed using the Kruskal–Wallis test and two-stage Benjamini, Krieger, and Yekutieli false discovery rate procedures (*p* > 0.05). The results were calculated from rarefied 20,000 reads per sample. The middle lines represent mean values.

**Figure 4:**
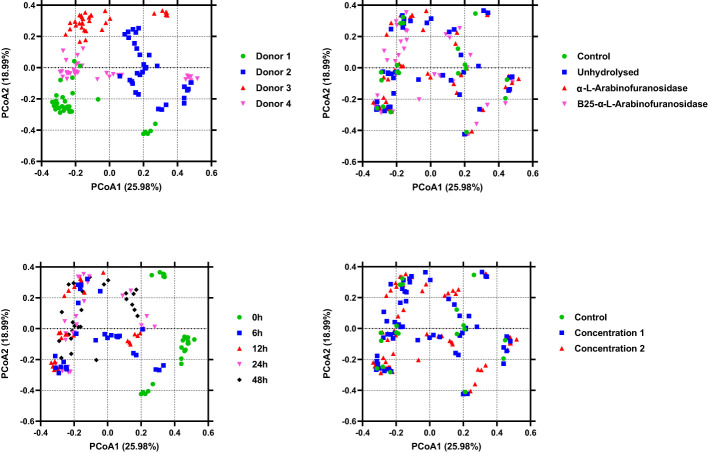
Plots of principal coordinate analysis among the identified microbiota in different samples show clustering based on the type of treatment, donor, and time. The samples are colored as indicated in the legends. Permutational multivariate analysis of variance and 999 permutations were used to assess the contributions of different factors to gut microbiota diversity.

The alpha diversity of the microbiota from *in vitro* fecal fermentation between 0 and 48 h was evaluated using Shannon entropy (**Figure 3**). No significant differences were found between the control samples and samples containing AX (*p* > 0.05). Nevertheless, the decrease in gut microbiota diversity following plate inoculation tended to be reversed faster in the untreated and AX groups than in the control group. The beta diversity across treatments, time, donor, and concentration was evaluated using PCoA based on Bray–Curtis distances. The results revealed that the microbial communities of the four donors differed from one another. The microbial communities were similar at 0 h, and showed a slight shift after 0 h. PCoA plots are shown in **Figure 4**.

### 3.2 Effects of wheat arabinoxylan on the gut microbiota composition

**Figure 5:**
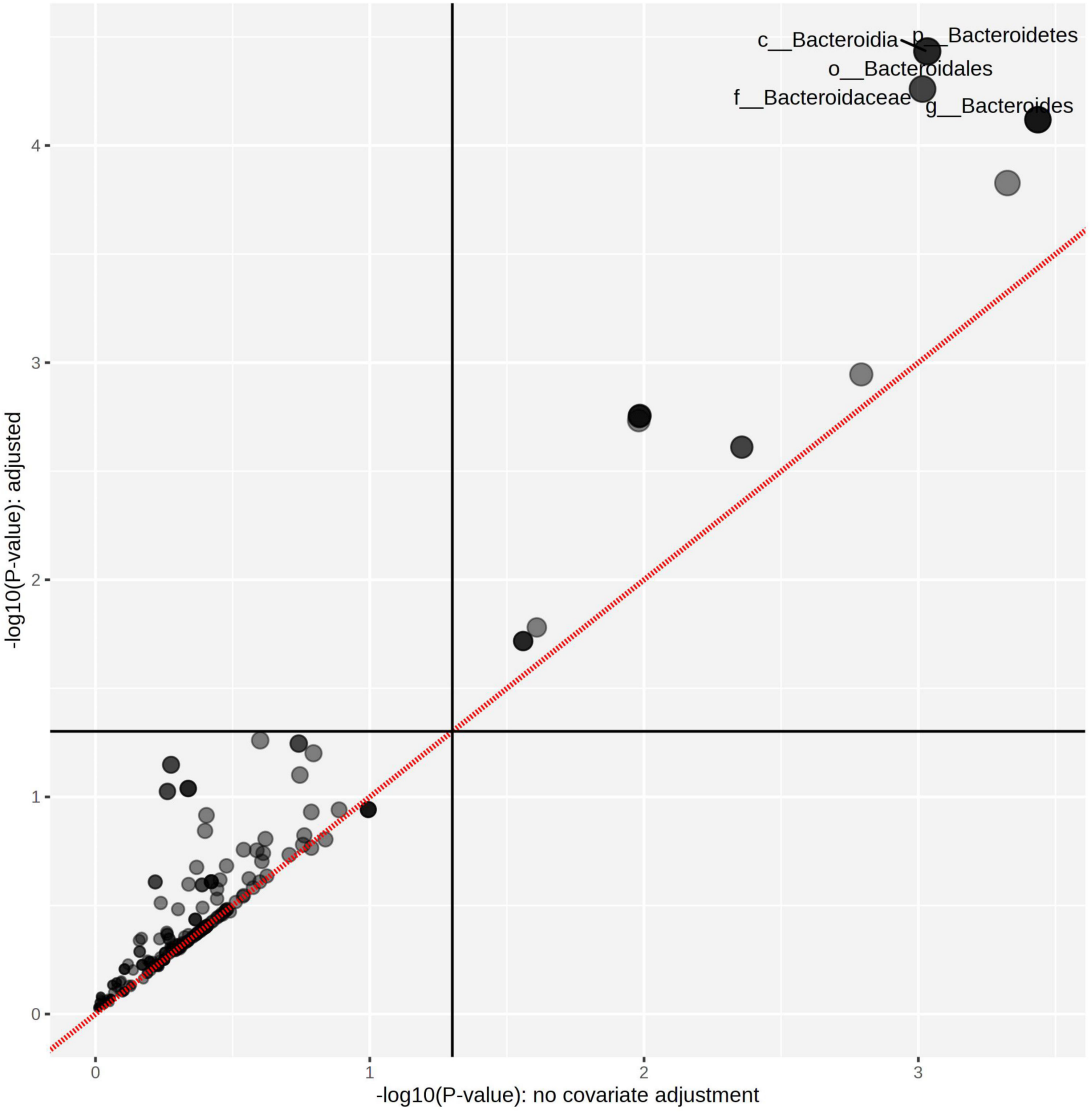
Treated arabinoxylan associated with significant differential taxa. The relative abundances of each taxon were summed, normalized, and autoscaled. A linear mixed model was then used to identify taxa with differential abundance by incorporating donor and time as confounding covariates.

**Figure 6:**
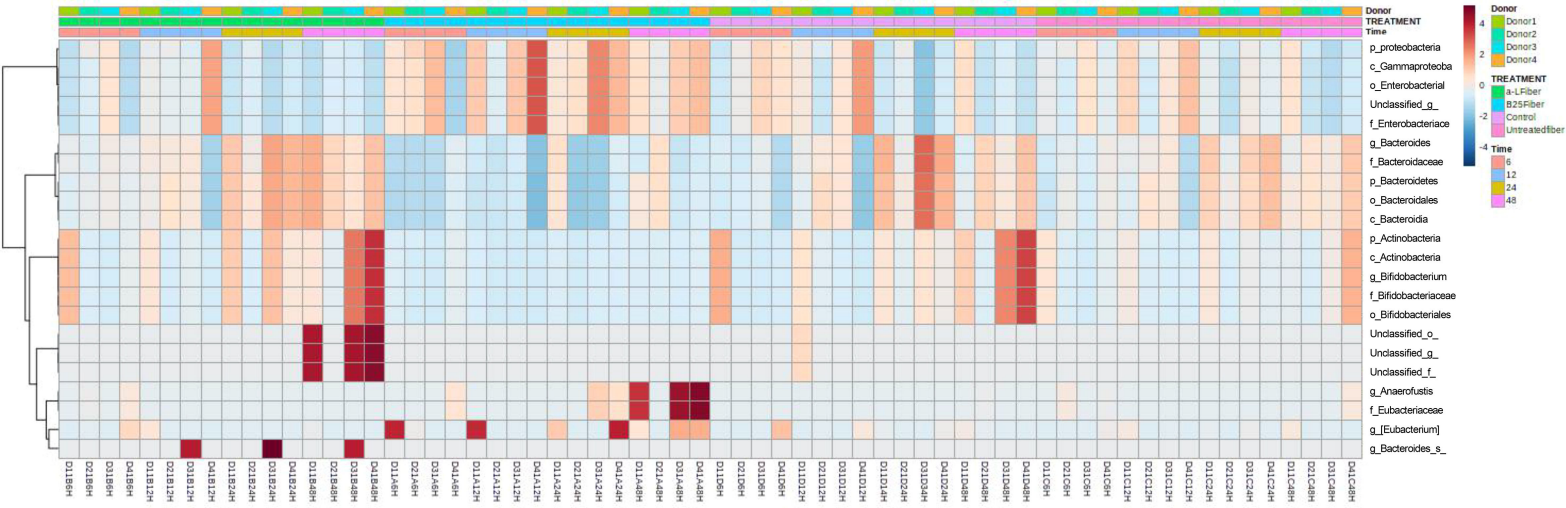
Heatmap showing the significant differential taxa identified by linear mixed model analysis, considering donor and time as covariates. The relative abundances of each taxon were summed, normalized, and autoscaled, and the autoscaled data were used to draw the heatmap and the scale shown. The samples were arranged by treatment, time, and donor.

**Figure 7:**
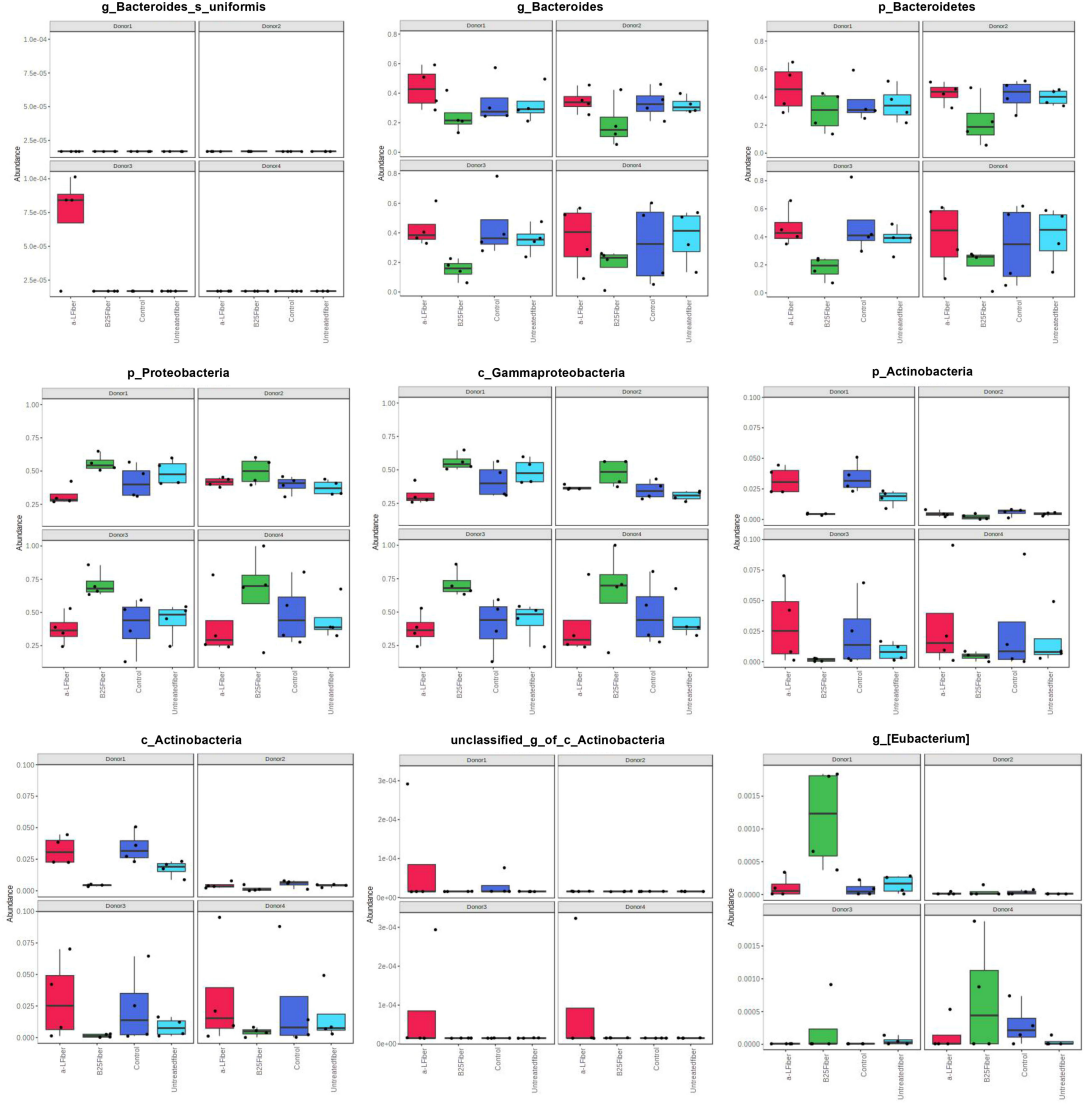
Histograms showing significant differential taxa identified by linear mixed model analysis. Each column and panel represent a treatment group and a donor, respectively. The relative abundances of each taxon were summed, normalized, and autoscaled. A linear mixed model was then used to identify taxa with differential abundance by incorporating donor and time as confounding covariates.

The linear mixed model analysis identified differential microbial taxa among the tested groups at various phylogenetic levels by adjusting for donor and time as covariates (**Figures 5**–**7**). Nine taxonomic features showed significant differences, specifically the *Bacteroidetes* phylum (**Figure 5**). Overall, 22 bacterial taxa belonging to *Proteobacteria*, *Bacteroidetes*, and *Actinobacteria* were significantly different at the phylum level (**Figure 6**). At the genus level, the α-L fibers increased the abundance of *Bacteroides* and *Bifidobacterium*. However, the B25-treated fiber improved the abundance of *Anaerofustis* and *Eubacterium* as compared with the control and untreated fiber groups. At the family level, *Enterobacteriaceae*, *Bifidobacteriaceae*, *Bacteroidaceae*, and *Eubacteriaceae* were the most differential taxa. *Proteobacteria*, *Bacteroidetes*, and *Actinobacteria* were more abundant at the phylum level in the treated and untreated AX groups than in the control group. **Figure 7** shows the effects of each AX fiber group on the microbiota composition shift and the significant bacterial taxa for each donor.

### 3.3 Effects of wheat arabinoxylan on gut microbiota metabolism

**Figure 8:**
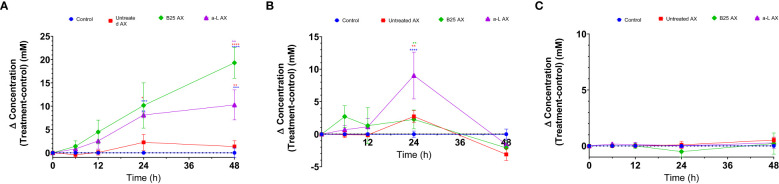
Concentration fold increase of short-chain fatty acids over the control for all donors (Δ concentration = test treatment − control) measured by gas chromatography over 48 h. Acetate, butyrate, and propionate **(A–C)**; control (circle, blue); B25 AX (diamond, green); untreated AX (square, red); α-L AX (triangle, purple). Statistical comparisons were conducted using repeated measures of two-way analysis of variance with Tukey’s multiple comparison test (*p* < 0.05). To determine significant differences, the samples were compared among themselves (*p < 0.05, **p < 0.01, ***p < 0.001, ****p < 0.0001).

**Figure 9:**
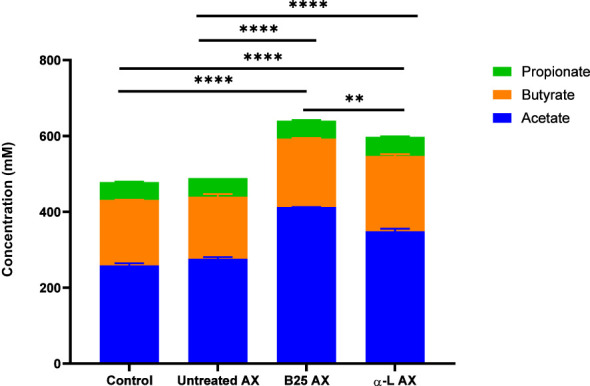
Concentration of total SCFAs from four healthy donors for each treatment. Data were analyzed using one-way analysis of variance (**p < 0.01, ****p < 0.0001).

The effect of wheat AX (treated and untreated) on SCFA generation was quantified using gas chromatography. In all treatment groups, acetate, butyrate, and propionate were the three major SCFAs produced by gut microbes (**Figure 8**). Bioengineered AX significantly increased the production of these metabolites (*p* < 0.05). For example, AX treated with the α-L-arabinofuranosidase B25 enzyme increased acetate production, and significantly differed from the control and untreated AX at 24 and 48 h. In comparison, AX treated with the α-L-arabinofuranosidase enzyme increased butyrate production, which was statistically significant at 24 h. For propionate, no significant differences were observed (*p* > 0.05) (**Figure 8C**). AX treated individually with α-L-arabinofuranosidase B25 and α-L-arabinofuranosidase enzymes produced the largest number of total SCFAs from all four donors and were significantly different from the untreated AX and control (**Figure 9**). The total number of SCFAs produced by both treated AXs were significantly larger than that of the untreated AX and control groups (*p* < 0.05).

## 4 Discussion

Numerous *in vitro, ex vivo*, and *in vivo* studies have investigated the modulatory effects of wheat AX and other prebiotic fibers on the gut microbiota. However, only a few studies have focused on the impact of structural modifications and the composition of wheat on the gut microbiota (Gong et al., 2019; Demuth et al., 2021). The dietary fiber structure is essential for determining its impact on gut microbiota composition. Studies have demonstrated that different microbial species have varying preferences for dietary fibers as substrates, primarily depending on the nature and structure of the fiber (Louis, 2017; Tuncil et al., 2017). Hence, we hypothesized that modifying the structure of wheat AX would have a differential effect on the gut microbiota composition by increasing the abundance of health-associated bacterial taxa and SCFA production. Demuth et al. investigated the effect of extruded and milled AX on the gut microbiome (Demuth et al., 2021), whereas Gong et al. studied the impact of whole and refined wheat on the gut microbiota (Gong et al., 2019). Our study aimed to understand better the structure–function relationship between wheat AX and the human gut microbiota. Here, we employed enzymatic digestion to modify the structure of wheat AX and produce two distinct groups of AX. AX treatment with α-L-arabinofuranosidase B25 was used to produce only L-arabinofuranosyl units on double-substituted β-D-xylopyranosyl, whereas treatment with α-L-arabinofuranosidase was used to produce only L-arabinofuranosyl units on single-substituted β-D-xylopyranosyl units. After that, the treated and untreated AX were subjected to *in vitro* fermentation with human colon microbiota from four healthy adults.

*Actinobacteria*, *Bacteroidetes*, and *Proteobacteria* were the predominant bacterial groups at the phylum level, which is consistent with previous studies (Wu et al., 2021; Mousavi et al., 2022). At the genus level, the treated AX fibers increased the abundance of *Bifidobacterium, Bacteroides, Anaerofustis*, and *Eubacterium.* The AX fiber treated with the α-L-arabinofuranosidase B25 enzyme enhanced the abundance of the genus *Anaerofustis*. *Anaerofustis* is considered a beneficial bacterial taxon owing to its association with butyrate production (Li et al., 2016). Butyrate is considered the most crucial SCFA because of its antidepressant and immunological properties (Smith et al., 2013). In addition, the enrichment of *Anaerofustis* with other dietary carbohydrates has been reported in several studies. For example, an animal study reported that the abundance *of Anaerofustis* increased in the presence of xylitol (Xiang et al., 2021). A similar result was observed in another study, which revealed that dietary supplementation with shredded steam-exploded pine particles (SSPPs) resulted in an increased abundance of *Anaerofustis* (Goel et al., 2022). The SSPPs used in the study were rich in carbohydrates, similar to the wheat AX used in our study. The population of *Anaerofustis* was reduced in patients with gastrointestinal diseases, such as inflammatory bowel disease, compared with their healthy counterparts (Masoodi et al., 2019). In addition, a recent study revealed that *Anaerofustis* is a bacterial taxon that is significantly negatively associated with anxiety (Li et al., 2022). The treated AX fibers were also enriched with *Eubacterium*. *Eubacterium* is a known dietary fiber fermenter and its presence in the gut is strongly correlated with increased dietary fiber consumption (Duncan et al., 2002; David et al., 2014). Furthermore, *Eubacterium* is a significant butyrate producer (Xiao et al., 2022), which is important for gut health, especially for controlling inflammation, managing immune reactions, and preserving gut barrier integrity (Mukherjee et al., 2020).

Furthermore, treating AX with α-L-arabinofuranosidase induced a bifidogenic effect by increasing the predominance of the *Bifidobacterium* genus. Likewise, Demuth et al. reported that wheat bran AX, which is structurally modified through milling and extrusion, rapidly improved the growth of *Bifidobacteria* compared with native AX after *in vitro* colonic fermentation (Demuth et al., 2021). Other studies have reported that wheat AX supports the growth of this bacterial group (Hughes et al., 2007; Paesani et al., 2019). *Bifidobacterium* is an important microorganism, and its presence has been linked to numerous therapeutic benefits and improved overall gastrointestinal health (Hidalgo-Cantabrana et al., 2017). For example, *Bifidobacterium* has been reported to be instrumental in lowering depressive-like symptoms, aiding the management of gut dysbiosis (Pinto-Sanchez et al., 2017; Tian et al., 2019; Tian et al., 2022), preserving intestinal barrier function, and protecting against pathogens (Rivière et al., 2016), and it exhibits anticancer properties by preventing the growth of colon cancer cells (Bahmani et al., 2019). Another *in vivo* study showed that the ingestion of wheat AX oligosaccharides (AXOS) significantly increased the abundance of *Bifidobacterium* in mice (Neyrinck et al., 2012). The study also postulated that the prebiotic and bifidogenic properties of AXOS were inversely related to obesity, inflammatory disorders, and gut barrier integrity (Neyrinck et al., 2012). Another study showed that wheat AX, when compared with inulin, promoted the growth of *Bifidobacterium* along with other beneficial microbes (Paesani et al., 2019). In addition, an *in vitro* study revealed that whole wheat and refined wheat after 24 h of fermentation increased the number of *Bifidobacterium* (Gong et al., 2019). Interestingly, we observed a significant enrichment of *Bacteroides uniformis* with the addition of AX treated with the α-L-arabinofuranosidase enzyme. Some studies have reported that certain dietary fibers promote the abundance of *B. uniformis*. Cantu-Jungles et al. demonstrated that the abundance of *B. uniformis* increased after the fecal fermentation of glucans from *Cookeina speciosa* (Cantu-Jungles et al., 2018). Similarly, insoluble β-glucan fibers have been reported to enhance the population of *B. uniformis* to over five times the initial population and increase butyrate production (Cantu-Jungles et al., 2021). Another study showed that pectin utilization by *B. uniformis* led to an increase in butyrate levels and the generation of gamma-aminobutyric acid (Benítez-Páez et al., 2017). These results concur with our observations, where the AX fiber treated with α-L-arabinofuranosidase enzyme supported the abundance of *B. uniforms* and increased butyrate production. Therefore, this might explain the high butyrate concentration in the α-L-arabinofuranosidase enzyme-treated AX fiber. In addition, the results of our study support the fibrolytic capabilities of *B. uniforms*. Finally, it is important to mention that the enzymes used in this study were from two bacterial species: α-L-arabinofuranosidase B25 was from *Bacteroides ovatus* and α-L-arabinofuranosidase was from *Bifidobacterium adolescentis*. We observed the abundance of these two microbial taxa after fermentation. For example, AX treated with α-L-arabinofuranosidase significantly enhanced the abundance of *Bifidobacterium* and *Bacteroides*. However, this is not surprising, as a recent study revealed that mice treated with *Bifidobacterium animalis* subsp*. lactis* XLTG11 had a greater abundance of *Bifidobacterium* and *Bacteroides* than the control group (Xu et al., 2022).

The production of SCFAs by the gut microbiota is essential because of their valuable effects. In addition to being the primary carbon source for gut microbes, they also maintain intestinal pH to support the growth of beneficial bacterial taxa (Putri et al., 2022). The impact of bioengineered AX on the metabolic activity of the gut microbiome was evident. All AX samples led to the production of major SCFAs, which is in agreement with previous studies involving AX (Paesani et al., 2020; Demuth et al., 2021; Gu et al., 2021). Of the three SCFAs, acetate and butyrate were the most abundant, whereas propionate was produced in a lower quantity than acetate and butyrate. This finding is consistent with those of previous studies (Mottawea et al., 2020; Tuncil et al., 2020; Gu et al., 2021; Mousavi et al., 2022). Nevertheless, the amount of SCFAs produced differed among the AX treatments. AX treated with the α-L-arabinofuranosidase B25 enzyme produced the greatest amount of total SCFAs, mainly acetate. In contrast, AX treated with α-L-arabinofuranosidase enzyme exhibited a significant butyrogenic effect by inducing the highest butyrate production at 24 h. The increased butyrate production by this group of treated AX could be related to the abundance of *Eubacterium* because the abundance of *Eubacterium* was induced by AX treated with the α-L-arabinofuranosidase enzyme. Butyrate is considered an essential SCFA for human health owing to its strong antidepressant potential (Hao et al., 2019). It is also the preferred source of energy for the intestinal epithelial cells (Smith et al., 2013; Rivière et al., 2016). The absence of butyrate has been linked to inflammatory bowel disease and colorectal cancer (Mottawea et al., 2016; Cresci et al., 2017). Mottawea et al. revealed that their synbiotic test formulation containing mainly polysaccharides, including tapioca fiber, lupin flour, and tiger-nut flour, substantially improved the amount of butyrate produced after 12 h of fermentation (Mottawea et al., 2020). Acetate is the most abundant SCFA that is produced during fermentation. This finding is consistent with the results of several studies (Fehlbaum et al., 2018; Gong et al., 2019; Mottawea et al., 2020; Demuth et al., 2021; Wu et al., 2021). Our results showed that treated AXs produced more acetate than the control and untreated AX. Specifically, AX treated with the α-L-arabinofuranosidase B25 enzyme induced the highest concentration of acetate production throughout the experiment. Interestingly, the acetate level increased exponentially toward the end of the experiment at 48 h. Acetate is an essential metabolite linked to various beneficial functions, such as modulating the release of colonic serotonin (Bhattarai et al., 2017) and, more recently, reducing cognitive decline (Zheng et al., 2021). Previous studies have reported an association between the presence of *Bifidobacteria* and acetate production (D’hoe et al., 2018; Gong et al., 2019). This may explain why we observed significantly high acetate levels between 24 and 48 h. The bifidogenic properties of AX are not surprising, as previously demonstrated (Paesani et al., 2019).

AX treated individually with α-L-arabinofuranosidase B25 and α-L-arabinofuranosidase produced the greatest amount of total SCFAs. The untreated and treated AX produced comparatively greater amounts of SCFAs than the control. More recently, Mio et al. reported similar results in their animal study. Their research showed that the fermentation of AX and β-glucan from barley resulted in increased production of total SCFAs compared with the control (Mio et al., 2022). Likewise, another recent study also revealed that agaro-oligosaccharide, produced by enzymatic hydrolysis, generated more SCFAs after 48 h fermentation, compared with the control and agaro-oligosaccharides from acid hydrolysis (Putri et al., 2022).

Indeed, the results of our research support the structure–function relationship between AX and the gut microbiome. For example, the hydrolysis of AX using α-L-arabinofuranosidase B25 enzyme derived from *Bacteroides ovatus* increased the abundance of *Anaerofustis* and *Eubacterium*. Conversely, AX hydrolyzed with α-L-arabinofuranosidase from *Bifidobacterium adolescentis* increased the abundance of *Bifidobacterium* and *Bacteroides*. Also, different AX structures induce differences in the production of SCFAs. Demuth et al. showed that extruded and milled wheat bran AX had differential effects on SCFA production after fermentation (Demuth et al., 2021). Finally, different bacterial groups selectively fermented treated and untreated AX. This finding may be attributed to the structure of the AX substrate because gut microbes have preferential substrates depending on factors such as the type of enzymes they produce and the sugar monomers they metabolize (Hughes et al., 2008; Louis, 2017).

## 5 Limitations

This *in vitro* study eliminated ethics concerns affecting prebiotic research *in vivo*/human studies. Nevertheless, this study also has limitations. The initial fecal microbiota was pre-cultured at pH 5.7. Maintaining the same pH in this batch fermentation model is complex and may affect the microbial diversity and activity. Also, the short fermentation time of 48 h fails to provide a view of long-term interactions between the AX substrate and gut microbes. Thus, it may be difficult to project long-term effects on the gut microbiota. As the fermentation process using AX fibers lasted only 48 h, we did not anticipate a strong and exceptional impact from the fiber fermentation. Generally, it takes approximately 3–5 days to achieve a diet-induced shift (David et al., 2014). In addition, the duration needed to notice a change in the gut microbiota following a dietary alteration is subject to variation based on multiple factors, including the particular type of dietary change, the person’s pre-existing gut microbiome, and other aspects of their lifestyle, such as exercise, stress levels, and medication usage (Zmora et al., 2019; Boytar et al., 2023). Furthermore, a rapid and pronounced shift in the gut microbiota following a brief dietary alteration may not necessarily be beneficial for an individual, as this suggests that even a small dietary change can significantly affect the gut microbiome. Notwithstanding, our test samples exhibited a more positive effect on gut microbiota composition and metabolism, and could potentially alleviate dysbiosis, which is worth noting.

## 6 Conclusion

Previous studies have focused on the prebiotic potential and effects of AX from different cereals on the gut microbiome for the treatment and management of obesity and other diseases. However, this study is among the first to investigate the structure–function relationship and impact of bioengineered AX on the human gut microbiome. The effect of bioengineered AX on the gut microbiota compared to untreated AX and the control, can be considered a prebiotic. In our study, bioengineered AX enhanced the abundance of *Roseburia inulinivorans* and *Bifidobacterium*, and reduced the availability of *Ruminococcaceae*. Similarly, both treated and untreated AX produced more metabolites than the control. These changes in the metabolism and composition of the gut microbiome indicate potential benefits to gut health. However, further comprehensive and long-term *in vivo* studies are recommended to strengthen the link between the structural modification of prebiotic fibers and overall gut health. Nevertheless, our research provides a scientific foundation for future studies involving the structural modification of dietary fibers. In addition, our research provided more evidence that the effect of dietary fibers on gut microbiome composition and metabolism can be structurally dependent by demonstrating how enzymatically bioengineered AX induces changes in the microbiota composition and metabolic profile. Finally, our research confirmed the prebiotic and bifidogenic properties of structurally modified AX, and supported the structure–function relationship between AX and the gut microbes. The results of this study also indicate that bioengineered AX selectively stimulates the growth of beneficial microorganisms, which concurs with the definition of a prebiotic.

## Data availability statement

The datasets presented in this study can be found in online repositories. The names of the repository/repositories and accession number(s) can be found *via* https://www.ncbi.nlm.nih.gov/, PRJNA759620.

## Ethics statement

The studies involving human participants were reviewed and approved by the University of Ottawa Research Ethics Board and Integrity. The patients/participants provided their written informed consent to participate in this study.

## Author contributions

Conceptualization: EN, WM, and RH. Methodology: EN, WM, and RH. Validation: EN, WM, and RH. Formal analysis: EN, HH, WM, and RH. Investigation: EN and WM. Resources: RH. Writing—original draft preparation—EN. Writing—review and editing—WM and RH. Visualization: EN, WM, and RH. Supervision: RH. Funding acquisition: RH. All authors contributed to the article and approved the submitted version.
